# “Specificity Determinants” Improve Therapeutic Indices of Two Antimicrobial Peptides Piscidin 1 and Dermaseptin S4 against the Gram-negative Pathogens *Acinetobacter baumannii* and *Pseudomonas aeruginosa*

**DOI:** 10.3390/ph7040366

**Published:** 2014-03-25

**Authors:** Ziqing Jiang, Adriana I. Vasil, Michael L. Vasil, Robert S. Hodges

**Affiliations:** 1 Department of Biochemistry & Molecular Genetics, University of Colorado, School of Medicine, Anschutz Medical Campus, Aurora, CO 80045, USA; E-Mail: ziqing.jiang@ucdenver.edu (Z.J.); 2 Department of Microbiology, University of Colorado, School of Medicine, Anschutz Medical Campus, Aurora, CO 80045, USA; E-Mails: adriana.vasil@ucdenver.edu (A.I.V.); mike.vasil@ucdenver.edu (M.L.V.)

**Keywords:** *Acinetobacter baumannii*, antimicrobial peptides, Gram-negative pathogens, *Pseudomonas aeruginosa*, specificity determinant(s), temperature-profiling by RP-HPLC

## Abstract

A new class of antimicrobial agents with lower rates of resistance and different targets is urgently needed because of the rapidly increasing resistance to classical antibiotics. Amphipathic cationic α-helical antimicrobial peptides (AMPs) represent such a class of compounds. In our previous studies, using a 26-residue *de novo* designed antimicrobial peptide, we proposed the concept of “specificity determinant(s)”: positively charged residue(s) in the center of the non-polar face of AMPs that could decrease hemolytic activity/toxicity but increase or maintain the same level of antimicrobial activity to increase dramatically the therapeutic index. In the current study, we used d-enantiomers of two AMPs, Piscidin 1 isolated from fish and dermaseptin S4 isolated from frog. We substituted different positions in the center of the hydrophobic face with one or two lysine residue(s) (one or two “specificity determinant(s)”). This simple modification not only maintained or improved antimicrobial activity against Gram-negative pathogens *Acinetobacter baumannii* (11 strains) and *Pseudomonas aeruginosa* (6 strains), but also dramatically decreased hemolytic activity of human red blood cells, as predicted. Therapeutic indices improved by 55-fold and 730-fold for piscidin 1 (I9K) and dermaseptin S4 (L7K, A14K), respectively, against *A. baumannii*. Similarly, the therapeutic indices improved 32-fold and 980-fold for piscidin 1 (I9K) and dermaseptin S4 (L7K, A14K), respectively, against *P. aeruginosa*.

## 1. Introduction

Emergence of antimicrobial resistance is becoming a very large public health threat and has been recognized by, amongst others, the World Health Organization [1], the U.S. Congress Office of Technology Assessment [2] and the United Kingdom House of Lords [3]. The urgency to develop new classes of antimicrobial agents against Gram-negative pathogens *Acinetobacter baumannii* and *Pseudomonas aeruginosa* was demonstrated by the dramatic increases in the incidence of antibiotic-resistant species in a recent study in Mexico [4]. In the latter study [4], 550 clinical isolates of *A. baumannii* and 250 clinical isolates of *P. aeruginosa* were analyzed for the prevalence of multi-drug resistance and 74% of *A. baumannii* and 34% of *P. aeruginosa* were multi-drug resistant. 

Antimicrobial peptides (AMPs) are widely distributed in nature and represent a promising class of new antimicrobial agents. AMPs are rapidly bactericidal and generally have broad-spectrum activity. It is difficult for bacteria to develop resistance to AMPs since their mode of action involves nonspecific interactions with the cytoplasmic membrane. In addition, enantiomeric forms of AMPs with all-d-amino acids have showed equal activities to their all-L-enantiomers [5,6,7,8,9,10,11,12,13], suggesting that the antimicrobial mechanism of such peptides does not involve a stereoselective interaction with a chiral enzyme or lipid or protein receptor. In addition, all d-peptides are resistant to proteolytic enzyme degradation, which enhances their potential as therapeutic agents. Moreover, it is relatively easy to engineer their structure, since peptide chemistry allows a multitude of time- and cost-effective modifications. However, it is widely believed that native AMPs lack specificity and might be too toxic (ability to lyse mammalian cells, normally expressed as hemolytic activity against human red blood cells) to be used for systemic treatment [14,15]. To overcome this problem, we developed the design concept of the “specificity determinant” which refers to substituting positively charged residue(s) in the center of the non-polar face of amphipathic α-helical or cyclic β-sheet antimicrobial peptides to create selectivity between eukaryotic and prokaryotic membranes; that is, antimicrobial activity is maintained and hemolytic activity or cell toxicity to mammalian cells is decreased or eliminated. Previously, we demonstrated that a hybrid peptide, V681 (cecropin A (1-8) + melittin B (1–18) derivative), had excellent antimicrobial activity, but also exhibited high toxicity to human red blood cells as measured by hemolytic activity [13,16,17]. We showed that a single valine to lysine substitution in the center of the non-polar face (V13K) dramatically reduced toxicity and increased the therapeutic index [13,16,17]. We recently addressed the question of whether we could take a broad-spectrum, 26-residue antimicrobial peptide in the all d-conformation with excellent biological properties and use a rational design approach to enhance further the biological properties if the focus was to develop a better Gram-negative AMP rather than maintain broad-spectrum activity. Our results clearly demonstrated the feasibility of such a proposal [17], where our final AMP had a 746-fold improvement (*i.e.*, decrease) in its hemolytic activity, maintained antimicrobial activity and improved the therapeutic indices by 1305-fold and 895-fold, respectively, against *A. baumannii* and *P. aeruginosa.*

In the current study, we chose piscidin 1 and dermaseptin S4 as two examples of native AMPs for substitution of one or two amino acid(s) to lysine(s) at different positions in the center of their non-polar faces to investigate the generality of the “specificity determinant” design concept to enhance or maintain antimicrobial activity and significantly improve the therapeutic index.

The dermaseptins are a family of linear peptides isolated from the skin of various tree-dwelling, South American frogs of the *Phyllomedusa* species were discovered in 1991 [18,19,20]. These amphipathic α-helical cationic antimicrobial peptides are structurally and functionally related. They exhibit rapid cytolytic activity against a variety of microorganisms including viruses [21,22], bacteria [23,24], protozoa [25], yeast and filamentous fungi [18,20]. Unlike other dermaseptin members, dermaseptin S4, a 28-residue AMP, lyses erythrocytes at micromolar concentrations. The HC_50_, the peptide concentration that causes 50% lysis of human red blood cells, was approximately 1.4 µM using a three hour incubation time at 37 °C [26]. Very rapid kinetics (within seconds) for the lysis of human red blood cells can be observed under a microscope [20,23,26].

The piscidin family comprises the most common group of AMPs in teleost fish [27]. Piscidin 1 was first isolated in 2001 from hybrid striped bass (*Morone saxatilis* male × *Morone chrysops* female), where it is produced in mast cells (immune cells of uncertain function present in all vertebrates) [28], skin, gill and gastrointestinal tract [29,30]. Piscidin 1 is a 22-residue amphipathic α-helical AMP rich in histidines and phenylalanines [28]. Piscidin 1 has the highest biological activity in this family with broad-spectrum activity against antibiotic-resistant bacteria, filamentous fungi, yeasts, and viruses [28,31,32]; however, piscidin 1 is not selective for bacterial *versus* mammalian cells, and caused hemolysis of human red blood cells with a HC_50_ of 11~20 µM, within one hour at 37 °C [33,34].

## 2. Experimental

### 2.1. Peptide Synthesis and Purification

Synthesis of the peptides was carried out by standard solid-phase peptide synthesis methodology using 9-fluorenylmethoxycarbonyl (Fmoc) chemistry and 4-methylbenzhydrylamine hydrochloride (MBHA) resin using a CEM Liberty microwave peptide synthesizer. The coupling procedure was catalyzed by 2-(1*H*-benzotriazol-1-yl)-1,1,3,3-tetramethyluronium hexafluorophosphate (HBTU, 0.5 M)/hydroxybenzotriazole (HOBt, 0.48 M) in dimethylformamide (DMF) with N,N-diisopropylethylamine (DIPEA, 2 M in N-methyl-2-pyrrolidinone (NMP)). The deprotection procedure (removal of Fmoc protecting group) was carried out by treatment of the resin with 0.1 M HOBt in DMF with 20% piperidine. After completion of the synthesis, the peptide resin was dried under vacuum and the peptide was cleaved from the resin with a mixture of 90% trifluoroacetic acid (TFA), 5% water and 5% triisopropylsilane (TIS) for 1–2 h. The resin was removed by filtration and peptide was precipitated with ice-cooled ethyl ether on ice for 1–2 h. The pellet was spun down and redissolved in acetonitrile/water (1:1, with 0.2% TFA) and the solution lyophilized to obtain the crude peptide. Peptide purification was performed by reversed-phase high-performance liquid chromatography (RP-HPLC) on a Zorbax 300 SB-C_8_ column (250 × 9.4 mm I.D.; 6.5 µm particle size, 300 Å pore size; Agilent Technologies, Little Falls, DE, USA) with a linear AB gradient at a flow rate of 2 mL/min, where eluent A was 0.2% aqueous TFA, pH 2, and eluent B was 0.18% TFA in acetonitrile. The crude sample was loaded onto the column in 0.2% aq. TFA, pH 2, followed by a 1% acetonitrile/min gradient to the point where a shallow 0.1% acetonitrile/min gradient started 12% below the acetonitrile concentration required to elute the peptide on injection of analytical sample using a gradient of 1% acetonitrile/min [35]. The 0.1% acetonitrile/min gradient was run for 170 min. Fractions of 4 mL were collected and fraction analyses on an analytical column (as described below) were carried out and the appropriate fractions were pooled and freeze-dried to obtain pure peptide.

### 2.2. Analytical RP-HPLC and Temperature Profiling of Peptides

The purity of the peptides was verified by analytical RP-HPLC and the peptides were characterized by mass spectrometry (LC/MS). Crude and purified peptides were analyzed on an Agilent 1100 series liquid chromatograph. Analytical runs were performed on a Zorbax 300 SB-C_8_ column (150 × 2.1 mm I.D.; 5 µm particle size, 300 Å pore size) from Agilent Technologies using a linear AB gradient (1% acetonitrile/min) and a flow rate of 0.25 mL/min, where eluent A was 0.2% aqueous TFA, pH 2, and eluent B was 0.18% TFA in acetonitrile. Temperature profiling analyses were performed on the same column in 3 °C increments, from 5 °C to 80 °C using a linear AB gradient of 0.5% acetonitrile/min, as described previously [13,16,17,36,37,38,39,40].

### 2.3. Characterization of Helical Structure

The mean residue molar ellipticities of peptides were determined by circular dichroism (CD) spectroscopy, using a Jasco J-815 spectropolarimeter (Jasco Inc. Easton, MD, USA) at 5 °C under benign (non-denaturing) conditions (50 mM NaH_2_PO_4_/Na_2_HPO_4_/100 mM KCl, pH 7.0), hereafter referred to as “benign buffer”, as well as in the presence of an α-helix inducing solvent, 2,2,2-trifluoroethanol, TFE, (50 mM NaH_2_PO_4_/Na_2_HPO_4_/100 mM KC1, pH 7.0 buffer/50% TFE). A 10-fold dilution of an approximately 500 µM stock solution of the peptide analogs was loaded into a 0.1 cm quartz cell and its ellipticity scanned from 195 to 250 nm. Peptide concentrations were determined by amino acid analysis.

### 2.4. Determination of Peptide Amphipathicity

Amphipathicity of peptides was determined by the calculation of hydrophobic moment [41], using the software package Jemboss version 1.2.1 [42], modified to include a hydrophobicity scale determined in our laboratory [43,44]. The hydrophobicity scale used in this study is as follows: Trp, 33.0; Phe, 30.1; Leu, 24.6; Ile, 22.8; Met, 17.3; Tyr, 16.0; Val, 15.0; Pro, 10.4; Cys, 9.1; His, 4.7; Ala, 4.1; Thr, 4.1; Arg, 4.1; Gln, 1.6; Ser, 1.2; Asn, 1.0; Gly, 0.0; Glu, −0.4; Asp, −0.8 and Lys, −2.0. These hydrophobicity coefficients were determined from RP-HPLC at pH 7 (10 mM PO_4_ buffer containing 50 mM NaCl) of a model random coil peptide with a single substitution of all 20 naturally occurring amino acids [43]. This HPLC-derived scale reflects the relative difference in hydophilicity/hydrophobicity of the 20 amino acid side-chains more accurately than previously determined scales (see recent review where this scale was compared to other scales [44]).

### 2.5. Gram-Negative Bacteria Strains Used in This Study

All the *A. baumannii* strains used in this study were (1) obtained from the collection of Dr. Anthony A. Campagnari at the University of Buffalo and originally isolated from different patients and organs/tissues (strain 649, blood; strain 689, groin; strain 759, gluteus; strain 821, urine; strain 884, axilla; strain 899, perineum; strain 964, throat; strain 985, pleural fluid and strain 1012, sputum); (2) were purchased from the American Type Culture Collection (ATCC, Manassas, VA, USA) (strain ATCC 17978, fatal meningitis; and strain ATCC 19606, urine).

*P. aeruginosa* strains used are as follows: strain PAO1 was isolated from a human wound in 1955 in Australia [45]; strain WR5 was isolated from a burn patient at Walter Reed Army Hospital, Washington, DC, USA, in 1976 and is a natural *toxA^−^* mutant, but is virulent in experimental mouse models [46,47]; strain PAK was originally isolated at Memorial University, St. John’s, Newfoundland, Canada, and is widely used in the analysis of pili [48,49]; strain PA14 was originally isolated as a clinical isolate in 1995 at the Massachusetts General Hospital, Boston, MA, USA and is virulent in a variety of plant and animal models of infection [50]; strain M2 was originally isolated in 1975 from the gastrointestinal tract of a healthy CF1 mouse, University of Cincinnati College of Medicine, and Shriners Burns Institute, Cincinnati, OH, USA and is virulent in a burn mouse model of *P. aeruginosa* infection [51]; and strain CP204 was isolated from a cystic fibrosis patient in 1989 at the National Jewish Medical and Research Center, Denver, CO, USA. All strains have been maintained at −80 °C in the laboratory of Michael Vasil, University of Colorado, School of Medicine.

### 2.6. Measurement of Antimicrobial Activity (MIC)

The minimal inhibitory concentration (MIC) is defined as the lowest peptide concentration that inhibited bacterial growth. MICs were measured by a standard microtiter dilution method in Mueller Hinton (MH) medium. Briefly, cells were grown overnight at 37 °C in MH broth and were diluted in the same medium. Serial dilutions of the peptides were added to the microtiter plates in a volume of 50 µL, followed by the addition of 50 µL of bacteria to give a final inoculum of 5 × 10^5^ colony-forming units (CFU)/mL. The plates were incubated at 37 °C for 24 h, and the MICs were determined.

### 2.7. Measurement of Hemolytic Activity (HC_50_)

Peptide samples (concentrations determined by amino acid analysis) were added to 1% human erythrocytes in phosphate-buffered saline (100 mM NaCl, 80 mM Na_2_HPO_4_, 20 mM NaH_2_PO_4_, pH 7.4) and the reaction mixtures were incubated at 37 °C for 18 h in microtiter plates. Two-fold serial dilutions of the peptide samples were carried out. Hemolytic activity was determined by withdrawing aliquots from the hemolysis assays and removing unlysed erythrocytes by centrifugation (800× *g*). Hemoglobin release was determined spectrophotometrically at 570 nm. The control for 100% hemolysis was a sample of erythrocytes treated with water or 0.1% Triton-X 100. The control for no release of hemoglobin was a sample of 1% erythrocytes without any peptide added. Since erythrocytes were in an isotonic medium, no detectable release (<1% of that released upon complete hemolysis) of hemoglobin was observed from this control during the course of the assay. The hemolytic activity was determined as the peptide concentration that caused 50% hemolysis of erythrocytes after 18 h (HC_50_). HC_50_ was determined from a plot of percent lysis *versus* peptide concentration (µM). We also determined the hemolytic activity after 1 hour at 37 °C.

### 2.8. Calculation of Therapeutic Index (HC_50_/MIC Ratio)

The therapeutic index is a widely accepted parameter to represent the specificity of antimicrobial peptides for prokaryotic *versus* eukaryotic cells. It is calculated by the ratio of HC_50_ (hemolytic activity) and MIC (antimicrobial activity); thus, larger values of therapeutic index indicate greater specificity for prokaryotic cells.

## 3. Results and Discussion

### 3.1. Peptide Design and Specificity Determinant(s)

**Figure 1:**
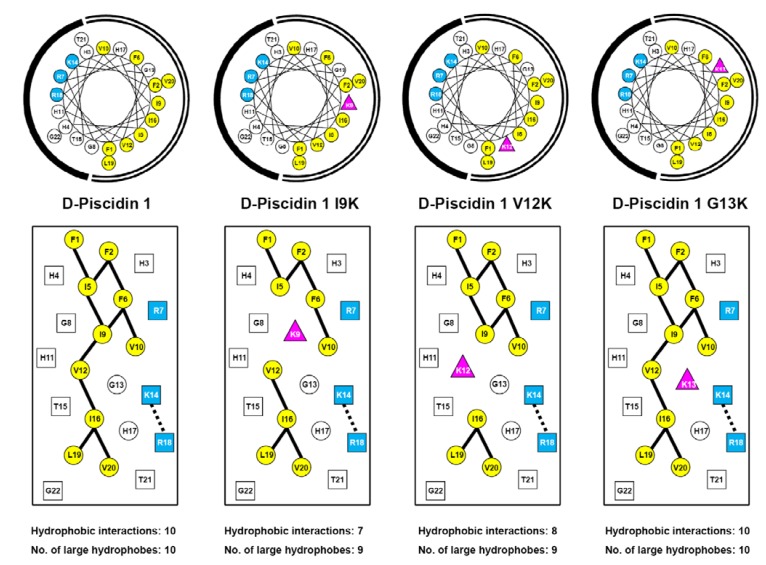
Helical wheel (upper panel) and helical net (lower panel) representation of d-piscidin 1 and analogs are shown in Table 1. The one-letter code is used for amino acid residues. d denotes that all residues in the peptides are in the d-conformation. Positively charged residues (Lys and Arg) are colored blue, large hydrophobic residues (Val, Ile, Leu and Phe) are colored yellow. The “specificity determinant” is denoted by pink triangles. In the helical wheel, the non-polar face is indicated as an open arc and the polar face is shown as a solid arc. In the helical net, the residues on the polar face are boxed and the residues on the non-polar face are circled. The *i*→*i*+3 and *i*→*i*+4 potential hydrophobic interactions along the helix are shown as black bars. The *i*→*i*+4 potential electrostatic repulsions between positively charged residues along the helix are shown as dotted bars.

**Figure 2:**
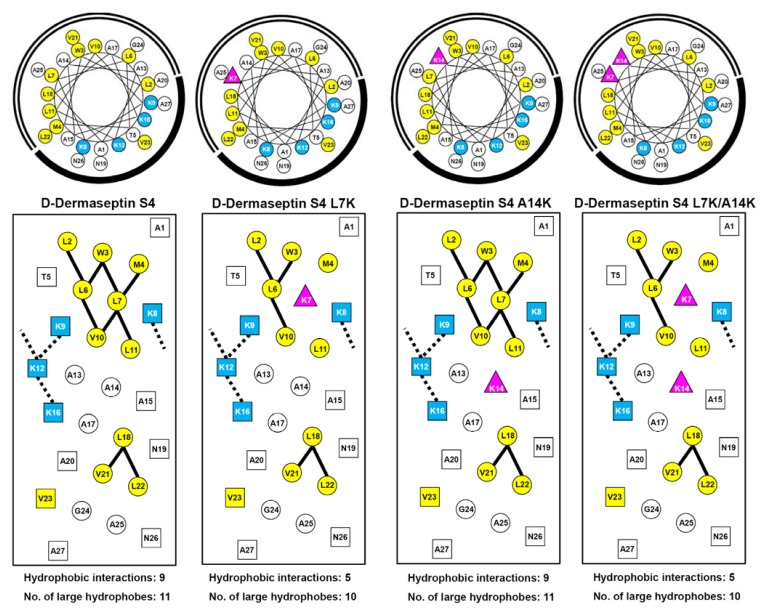
Helical wheel (upper panel) and helical net (lower panel) representation of d-dermaseptin S4 and analogs are shown in Table 1. The one-letter code is used for amino acid residues. d denotes that all residues in the peptides are in the d conformation. Positively charged residues (Lys) are colored blue, large hydrophobic residues (Val, Leu, Met and Trp) are colored yellow. The “specificity determinants” are denoted by pink triangle(s). In the helical wheel, the non-polar face is indicated as an open arc and the polar face is shown as a solid arc. In the helical net, the residues on the polar face are boxed and the residues on the non-polar face are circled. The *i*→*i*+3 and *i*→*i*+4 potential hydrophobic interactions along the helix are shown as black bars. The *i*→*i*+3 and *i*→*i*+4 potential electrostatic repulsions between positively charged residues along the helix are shown as dotted bars.

As mentioned above, enantiomeric forms of AMPs with all-d-amino acids have showed equal activities to their all-l-enantiomers. The property that all d-peptides are resistant to proteolytic enzyme degradation enhances their potential as therapeutic agents. In this study, we designed and synthesized nine all-d antimicrobial peptides including the native sequences, d-piscidin 1 and a 27-residue version of d-dermaseptin S4 which has a deletion of Ala18. This included four analogs of d-piscidin 1 and three analogs of d-dermaseptin S4 as shown in Table 1. Figure 1 and Figure 2 show the amino acid sequences in helical wheel and helical net representations. The positively charged lysine or arginine residues are colored blue and are located on the polar face of the AMP. The large hydrophobes (Val, Ile, Leu, Met, Phe and Trp) are colored yellow and are located on the non-polar face of the AMP. The only exception is V23 in dermaseptin S4 which is located in the center of the polar face. However, there is only one large hydrophobe on the polar face compared to ten large hydrophobes on the non-polar face. The positively charged residue(s) in the center of the non-polar face (“specificity determinant(s)”) are denoted as pink triangles. The potential *i* to *i*+3 or *i* to *i*+4 electrostatic repulsions between positively charged residues are shown as black dotted lines. The *i* to i+3 or *i* to *i*+4 hydrophobic interactions between large hydrophobes are shown as solid black lines. These representations allow easy comparison of different analogs to explain their biological and biophysical properties describe below.

The design concept of “specificity determinant(s)” (positively charged lysine residue(s) in the center of non-polar face of α-helical AMPs) introduced by our laboratory previously achieved the following biophysical and biological properties: (i) decreased the number of hydrophobic interactions and disrupted the continuous hydrophobic surface that stabilizes the helical structure of the AMP; (ii) reduced the hydrophobicity on the non-polar face and overall hydrophobicity; (iii) prevented peptide self-association in aqueous conditions [16,17]; (iv) dramatically reduced hemolytic activity; (v) maintained or enhanced antimicrobial activity and (vi) dramatically improved the therapeutic index [16,17]. The “specificity determinant” allowed our antimicrobial peptides to discriminate between eukaryotic and prokaryotic cell membranes, that is, exhibit pronounced selectivity for prokaryotic cell membranes. The use of our approach has also been recently validated by another group who demonstrated the importance of a positively charged residue in the non-polar face (Leu to Arg) of a native 16-residue antimicrobial peptide, RTA3 (derived from *Streptococcus mitis*), which decreased hemolytic activity by 20-fold [52].

**Table 1:** Peptides used in this study.

Peptide Name ^a^	Length	Sequence ^b^	MW
D-Piscidin 1	22	NH_2_-FFHHIFRGIVHVGKTIHRLVTG-amide	2571
D-Piscidin 1 G8P ^c^	22	NH_2_-FFHHIFRPIVHVGKTIHRLVTG-amide	2611
D-Piscidin 1 I9K	22	NH_2_-FFHHIFRG**K**VHVGKTIHRLVTG-amide	2586
D-Piscidin 1 V12K	22	NH_2_-FFHHIFRGIVH**K**GKTIHRLVTG-amide	2600
D-Piscidin 1 G13K	22	NH_2_-FFHHIFRGIVHV**K**KTIHRLVTG-amide	2642
D-Dermaseptin S4	27	NH_2_-ALWMTLLKKVLKAAAKALNAVLVGANA-amide	2778
D-Dermaseptin S4 L7K	27	NH_2_-ALWMTL**K**KKVLKAAAKALNAVLVGANA-amide	2794
D-Dermaseptin S4 A14K	27	NH_2_-ALWMTLLKKVLKA**K**AKALNAVLVGANA-amide	2837
D-Dermaseptin S4 L7K, A14K	27	NH_2_-ALWMTL**K**KKVLKA**K**AKALNAVLVGANA-amide	2851
Control C ^d^	18	Ac-ELEKGGLEGEKGGKELEK-amide	-

^a^ The d- denotes that all amino acid residues in each peptide are in the d conformation; ^b^ Peptide sequences are shown using the one-letter code for amino acid residues; Ac denotes *N*^α^-acetyl and amide denotes *C*^α^- amide. The “specificity determinant(s)”, Lys residues incorporated in the center of the non-polar face, are bolded; ^c^ Results of l-piscidin 1 G8P were published previously by Lee *et al.*, [33] and was their most selective peptide; ^d^ This peptide is a random coil peptide in the all lconformation used as a control for reversed-phase chromatography temperature profiling to examine peptide self-association.

In the current study, α-helical d-piscidin 1 and d-dermaseptin S4 are used as frameworks to substitute one or two lysine residue(s) at different positions in the center of the non-polar face (I9K, V12K and G13K for d-piscidin 1 (Figure 1) and L7K, A14K and L7K,A14K for d-dermaseptin S4 (Figure 2) to investigate the effect of such “specificity determinant(s)” on their biophysical properties including hydrophobicity, amphipathicity, helicity and self-association (oligomerization) as well as their biological activities including antibacterial activities against six strains of *P. aeruginosa* and eleven strains of *A. baumannii*, hemolytic activities to human red blood cells and therapeutic indices. 

### 3.2. Peptide Hydrophobicity

Amphipathic α-helical AMPs must have a certain minimum hydrophobicity to penetrate the hydrophobic membrane of prokaryotic cells. Hydrophobicity is one of the design features to optimize in AMPs. It is generally accepted that increasing the hydrophobicity of the non-polar face of amphipathic α-helical AMPs will increase antimicrobial activity. However, our laboratory made a major contribution to understanding the role of hydrophobicity in antimicrobial and hemolytic activity [37]. At relatively low levels of hydrophobicity on the non-polar face, an increase in peptide hydrophobicity caused an improvement in antimicrobial activity until an optimum hydrophobicity was reached, at which point further increases in hydrophobicity beyond this optimum resulted in a dramatic loss of antimicrobial activity. In other words, there is an optimal hydrophobicity window where decreases or increases in hydrophobicity outside this window cause significant decreases in antimicrobial activity [37].

However, this relationship is not observed with hemolytic activity, where increasing hydrophobicity correlates with stronger hemolytic activity and no decrease in hemolytic activity is observed at high hydrophobicity, where antimicrobial activity is dramatically decreased. We have associated the decrease in antimicrobial activity with high hydrophobicity and strong peptide self-association, which prevents the AMP from passing through a capsule or the cell wall in prokaryotic cells to reach the cytoplasmic membrane. Peptide dimers/oligomers are in their folded α-helical conformation and would be inhibited from passing through a capsule and a cell wall to reach the target membranes. There is are no polysaccharide-based cell walls in eukaryotic cells, thus increasing hydrophobicity usually increases hemolytic activity on human red blood cells. Our “specificity determinant(s)” disrupt α-helical structure in aqueous media and maintain an unfolded monomer which can more easily penetrate a capsule and cell wall of prokaryotic cells to reach the membrane where the hydrophobicity of the membrane induces peptide folding into an α-helical structure and the AMP can now disrupt the membrane causing leakage and cell death [37]. Thus, there is an optimum hydrophobicity for each AMP to have the best antimicrobial activity and the least hemolytic activity.

In the current study, substituting a large hydrophobic residues, isoleucine or valine (I9K or V12K), in the center of the non-polar face of d-piscidin 1 dramatically reduced overall hydrophobicity (more than 10 min as measured by RP-HPLC retention time (Table 2)) and disrupted two (for d-piscidin 1 V12K) or three (for d-piscidin 1 I9K) hydrophobic interactions between large hydrophobic residues that stabilize the hydrophobic surface of the helix (Figure 1). However, switching the hydrophilic glycine to lysine at position 13 (d-piscidin G13K) had very little effect on overall hydrophobicity. The retention times were 76.4 min and 74.6 min for d-piscidin 1 and d-piscidin 1 G13K, respectively (Table 2). For d-dermaseptin S4, a single leucine to lysine substitution at position 7 disrupted four hydrophobic interactions on the non-polar face (Figure 2), thus decreasing peptide overall hydrophobicity by more than 29 min (Table 2). A second “specificity determinant” at position 14 (d-dermaseptin S4 L7K, A14K) further decreased hydrophobicity by almost 17 min, that is, a total decrease of 46 min from native d-dermaseptin S4 (Table 2).

**Table 2:** Biophysical data.

Peptide name	Net charge	Hydrophobicity	Benign		50% TFE		P_A_^d^	Amphipathicity ^e^
		t_R_^a^ (min)	[θ]_222_^b^	%Helix ^c^	[θ]_222_^b^	%Helix ^c^		
d-Piscidin 1	+3	76.4	100	<1	36,200	100	0.78	5.32
d-Piscidin 1 I9K	+4	65.4	−300	<1	20,950	58	1.29	4.24
d-Piscidin 1 V12K	+4	65.9	−200	<1	16,000	44	0.69	4.81
d-Piscidin 1 G13K	+4	74.6	−250	<1	34,050	94	0.95	5.27
d-Dermaseptin S4	+4	124.4	28,900	75	38,400	100	12.61	3.58 (5.48)
d-Dermaseptin S4 L7K	+5	95.1	1,950	5	27,250	71	4.80	2.64 (4.12)
d-Dermaseptin S4 L7K,A14K	+6	78.6	2360	6	36,042	94	2.29	2.42 (3.76)

^a^ t_R_ denotes retention time in RP-HPLC at pH 2 and room temperature, and is a measure of overall peptide hydrophobicity; ^b^ The mean residue molar ellipticities [**θ**]_222_ (deg cm^2^/dmol) at wavelength 222 nm were measured at 5 °C in benign conditions (100 mM KCl, 50 mM NaH_2_PO_4_/Na_2_HPO_4_, pH 7.0) or in benign buffer containing 50% trifluoroethanol (TFE) by circular dichroism spectroscopy; ^c^ The helical content (as a percentage) of a peptide relative to the molar ellipticity value of parent peptide (d-piscidin 1 or d-dermaseptin S4) in the presence of 50% TFE; ^d^ P_A_ denotes self-association parameter (dimerization/oligomerization) of each peptide during RP-HPLC temperature profiling, which is the maximal retention time difference of (t_R_^t^-t_R_^5^ for peptide analogs)-(t_R_^t^-t_R_^5^ for control peptide C) within the temperature range; t_R_^t^-t_R_^5^ is the retention time difference of a peptide at a specific temperature (t_R_^t^) compared with that at 5 °C (t_R_^5^). The sequence of the random coil control peptide C is shown in Table 1; ^e^ Amphipathicity was determined by calculation of hydrophobic moment [41] using hydrophobicity coefficients determined by RP-HPLC [43,44]; see methods for details. The amphipathicity values for d-dermaseptin S4 and its analogs (residues 1–14) are shown in brackets.

### 3.3. Amphipathicity

**Figure 3:**
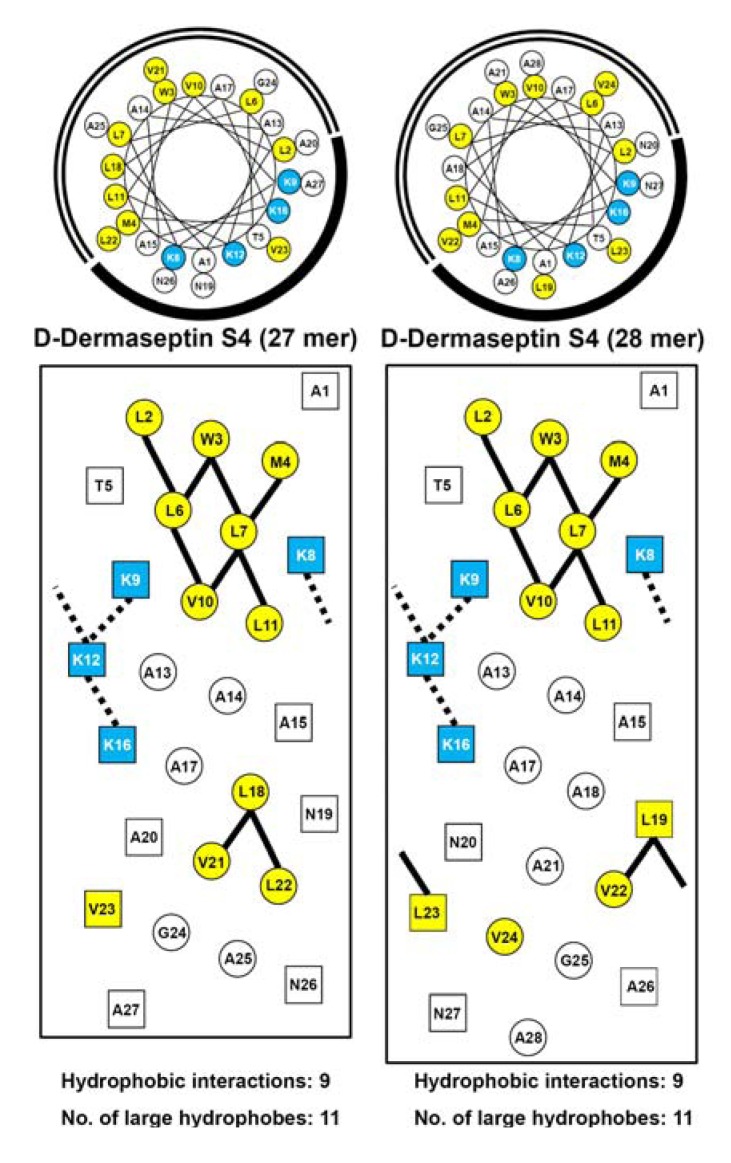
Helical wheel (upper panel) and helical net (lower panel) representation of d-dermaseptin S4 (27 mer) and d-dermaseptin S4 (28 mer). The one-letter code is used for amino acid residues. d denotes that all residues in the peptides are in the d conformation. Positively charged residues (Lys) are colored blue, large hydrophobic residues (Val, Leu, Met and Trp) are colored yellow. In the helical wheel, the non-polar face is indicated as an open arc and the polar face is shown as a solid arc. In the helical net, the residues on the polar face are boxed and the residues on the non-polar face are circled. The *i*→*i*+3 and *i*→*i*+4 potential hydrophobic interactions along the helix are shown as black bars. The *i*→*i*+3 and *i*→*i*+4 potential electrostatic repulsions between positively charged residues along the helix are shown as dotted bars.

The native sequence of d-piscidin 1 is very amphipathic with a value of 5.32 (Table 2). d-piscidin 1 G13K, the analog where there was no loss of a hydrophobe on substitution of a lysine residue on the non-polar face, maintained the same level of amphipathicity with a value of 5.27. Substituting one large hydrophobe with lysine, lowered the amphipathicity of d-piscidin 1 V12K and d-piscidin 1 I9K to 4.81 and 4.24, respectively. However, these analogs with the single specificity determinant still remain very amphipathic. Figure 3 shows a comparison of the 27- and 28-residue version of d-dermaseptin S4. Of particular interest is that the deletion of Ala18 from the 28-residue sequence dramatically changes the composition of the non-polar face. In the case of d-dermaseptin S4 (28 mer) two hydrophobic Leu19 and Leu23 residues are located in the center of the polar face (Figure 3, right panel), whereas in the case of d-dermaseptin S4 (27 mer) only a single hydrophobe, Val23, remains located in the center of the polar face (Figure 3, left panel). This subtle change also has a large effect on the amphipathicity of the AMP. The amphipathicity values for d-dermaseptin S4 (27 mer) and (28 mer) are 3.58 and 1.63, respectively. Due to this large difference and the fact that most AMPs are highly amphipathic we decided to investigate the biological and biophysical properties of the 27-residue version of d-dermaseptin S4. Substituting with one or two lysine residues at positions 7 and 14 lowered the amphipathicity to 2.64 for d-dermaseptin S4 L7K and 2.42 for d-dermaseptin S4 L7K, A14K. It should be noted that, although the overall amphipathicity is low, the helical region identified in the NMR studies [53] (residues 1-14) has an amphipathicity value of 5.48 for native d-dermaseptin S4 (1-14), 4.12 for d-dermaseptin S4 (1–14) L7K and 3.76 for d-dermaseptin S4 (1–14) L7K, A14K (Table 2). The amphipathicity of region 1–14 can explain the overall hydrophobicity of d-dermaseptin S4 and its analogs. The specificity determinant(s) in the non-polar face decreased overall hydrophobicity and amphipathicity as expected but these molecules remained very amphipathic when in a helical conformation.

### 3.4. Secondary Structure of Peptides

**Figure 4:**
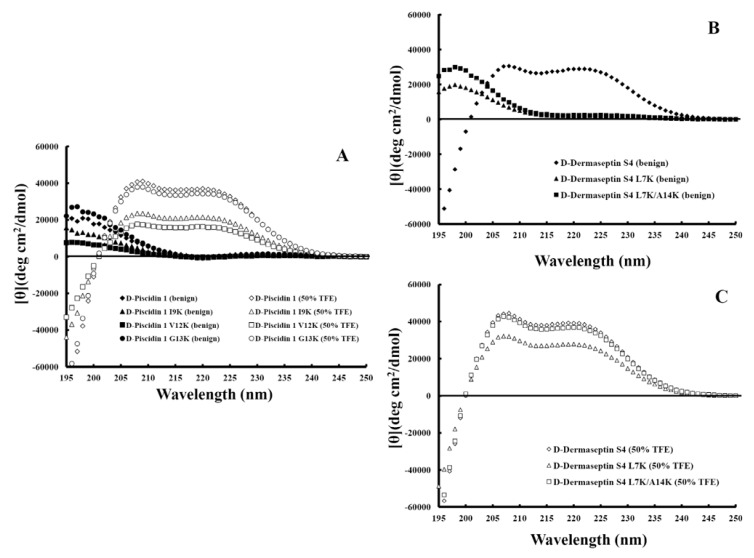
Panel **A** shows the CD spectra of d-pisicidin 1 analogs in aqueous benign buffer (100 mM KCl, 50 mM NaH_2_PO_4_/Na_2_HPO_4_ at pH 7.0), 5 °C (closed symbols) and in the presence of buffer-trifluoroethanol (TFE) (1:1, *v*/*v*) (open symbols). Panel **B** shows the CD spectra of d-dermaseptin S4 analogs in aqueous benign buffer (100 mM KCl, 50 mM NaH_2_PO_4_/Na_2_HPO_4_ at pH 7.0), 5 °C and panel **C** shows d-dermaseptin S4 analogs in the presence of buffer-trifluoroethanol (TFE) (1:1, *v*/*v*).

Figure 4 shows the CD spectra of the peptides in different environments (*i.e.*, under benign conditions (non-denaturing) and in the buffer with 50% TFE (to mimic the hydrophobic environment of the membrane). It should be noted that all-D α-helical peptides exhibited a positive spectrum [13]. The helicities of the peptides in benign buffer and in 50% TFE relative to that of their native peptide (d-piscidin 1 or d-dermaseptin S4) in 50% TFE were determined (Table 2). All d-piscidin 1 analogs showed negligible secondary structure in benign buffer (Figure 4A closed symbols) and a typical α-helix spectrum with double maxima at 208 and 222 nm in the non-polar environment of 50% TFE, a mimic of hydrophobicity and the α-helix-inducing ability of the membrane (Figure 4A open symbols). d-piscidin 1 I9K and d-piscidin 1 V12K, the analogs with one large hydrophobe in the non-polar face replaced by lysine, showed, respectively, a 42% and 56% decrease in helicity in 50% TFE, while d-piscidin 1 G13K, the analog where there was no loss of a hydrophobe on substitution of a lysine residue, showed only 6% reduction in helicity compared to that of the native sequence (Table 2). d-dermaseptin S4 was 75% α-helical in benign medium and was completely induced to α-helical structure in the presence of 50% TFE. By comparison, the analogs d-dermaseptin S4 L7K and d-dermaseptin S4 L7K, A14K showed no α-helical structure in benign medium, indicating that lysine substitutions on the hydrophobic face completely disrupted α-helical structure (Figure 4B). Helical structure can be induced in these two analogs in the presence of a hydrophobic environment (Figure 4C).

The general effect of “specificity determinant(s)” is to reduce or remove α-helical structure in benign media but allow induction of α-helical structure in the present of the hydrophobicity of the membrane.

### 3.5. Peptide Self-Association

Peptide self-association (*i.e.*, the ability to oligomerize / dimerize) in aqueous solution is a very important parameter for antimicrobial activity. We assume that monomeric random-coil antimicrobial peptides are best suited to pass through a polysaccharide capsule, the outer membrane (*i.e.*, lipopolysaccharide), and the cell wall (*i.e.*, peptidoglycan) of microorganisms prior to penetration into the cytoplasmic membrane, induction of α-helical structure and disruption of membrane structure to kill target cells. Thus, if the self-association ability of a peptide in aqueous media is too strong (e.g., forming stable folded dimers/oligomers through interaction of their non-polar faces) this could decrease the ability of the peptide to dissociate to monomer where the dimer/oligomer cannot effectively pass through the capsule and cell wall to reach the membrane. The ability of the peptides in the present study to self-associate was determined by the technique of RP-HPLC temperature profiling at pH 2 over the temperature range of 5 °C to 80 °C. This novel method to measure self-association of small amphipathic cyclic β-sheet molecules was first reported by Lee and coworkers in 2003 [36]. The utility of the method was then applied to the monitoring of dimerization and unfolding of *de novo* designed synthetic α-helical peptides [54], the folding and stability of two-stranded α-helical coiled-coils [55] and, finally, as a key method in the design and optimization of amphipathic α-helical antimicrobial peptides (AMPs) [13,16,17,37,38,39,40]. 

**Figure 5:**
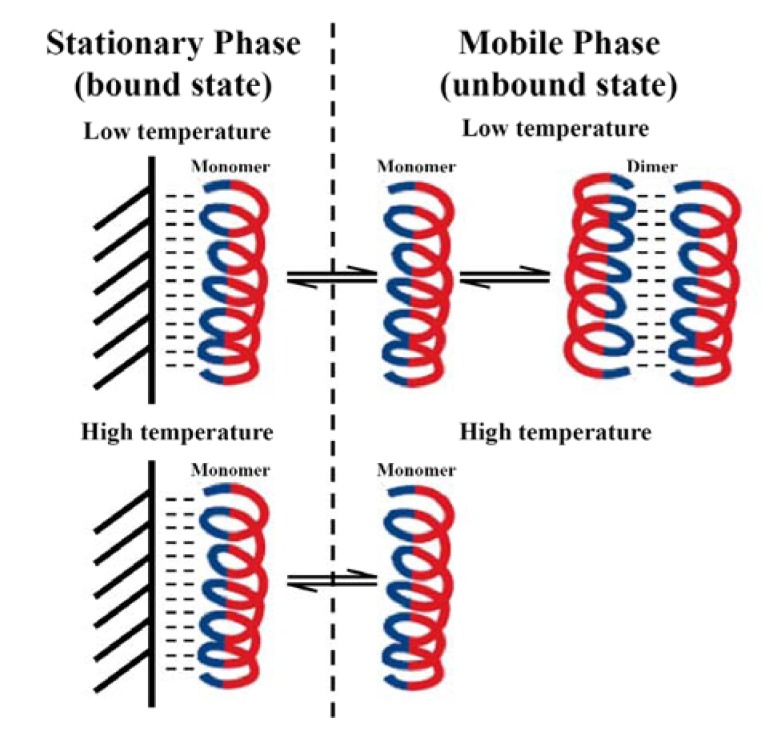
Proposed mechanism for temperature profiling by RP-HPLC. Only the folded monomeric α-helix is bound to the hydrophobic reversed-phase matrix. During partitioning at low temperature, there is an equilibrium between monomer and dimer in the mobile phase. At some higher temperature during partitioning there is only monomer present in the mobile phase. This method measures the self-association parameter for any amphipathic molecule as shown for d-dermaseptin S4 and its analogs in Figure 6.

To optimize improvement in the biological properties of AMPs, it is important to understand how the RP-HPLC temperature profiling method works. At low temperature, AMPs are capable of self-associating in aqueous solution via their non-polar faces. In the case of dimerization, equilibrium is established between monomer and dimer and the concentration of monomer and dimer at any given temperature depends on the strength of the hydrophobic interaction between the two monomers. In RP-HPLC, the hydrophobicity of the matrix disrupts/dissociates the dimer and only the monomeric form of the peptide is bound to the hydrophobic matrix by its non-polar face (preferred binding domain) (Figure 5). Only the monomeric form of the AMP can partition between the alkyl ligands on the reversed-phase column and the mobile phase. At low temperature, the monomer can dimerize in the mobile phase and the retention time is decreased due to the large population of dimers in solution. At higher temperatures, the population of dimers in the mobile phase during partitioning decreases, thereby increasing the concentration of monomeric peptide in solution and thereby increasing retention time. At some higher temperature no dimer exists in the mobile phase and the peptide has the maximum retention time. With a random coil peptide that does not dimerize, the peptide binds to the stationary phase and partitions in the mobile phase as a monomer with undefined structure throughout the temperature range of 5 °C to 80 °C. Thus, the effect of temperature on retention time is linear and decreases with increasing temperature.

**Figure 6:**
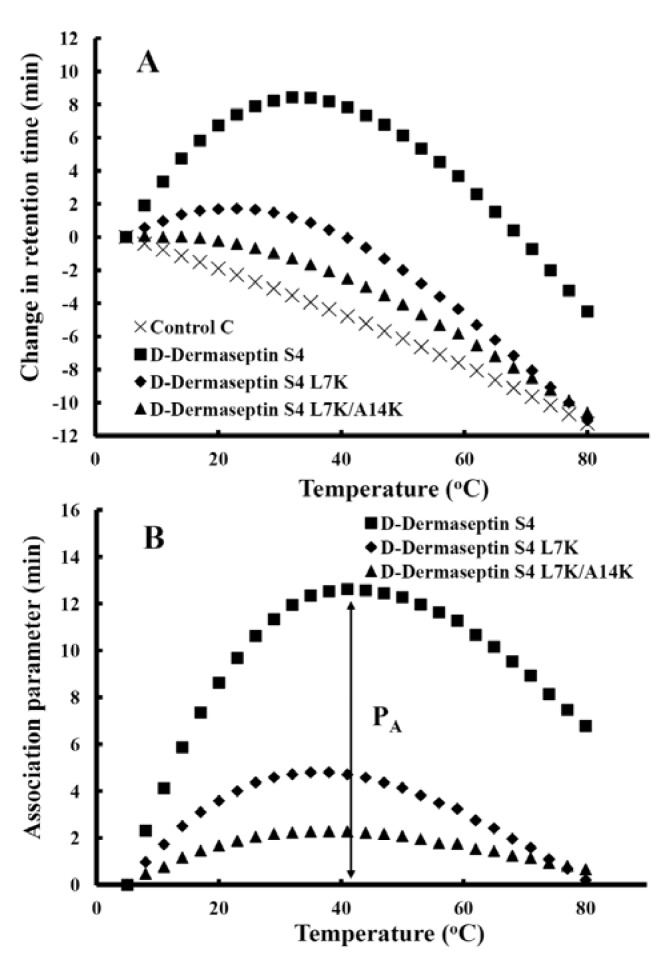
d-dermaseptin S4 analogs self-association ability as monitored by temperature profiling in RP-HPLC. In panel **A**, the retention times of peptides are normalized to 5 °C through the expression (t_R_^t^–t_R_^5^), where t_R_^t^ is the retention time at a specific temperature of an antimicrobial peptide or control peptide C, and t_R_^5^ is the retention time at 5 °C. In panel **B**, the retention behavior of the peptides was normalized to that of control peptide C through the expression (t_R_^t^–t_R_^5^ for peptides)–(t_R_^t^–t_R_^5^ for control peptide C). The maximum change in retention time from the control peptide C defines the peptide association parameter, denoted P_A_ (Table 2). The sequences of the peptides and the random coil control peptide are shown in Table 1.

Figure 6A shows the retention behavior of d-dermaseptin S4 and its peptide analogs after normalization to their retention times at 5 °C. Control peptide C shows a linear decrease in retention time with increasing temperature and is representative of peptides which have no ability to self-associate during RP-HPLC. Control peptide C is a monomeric random coil peptide in both aqueous and hydrophobic media; thus, its linear decrease in peptide retention behavior with increasing temperature within the range of 5 °C to 80 °C represents only the general effects of temperature due to greater solute diffusivity and enhanced mass transfer between the stationary and mobile phase at higher temperatures. To allow for these general temperature effects, the data for the control peptide was subtracted from each temperature profile, as shown in Figure 6B. Thus, the peptide self-association parameter, P_A_, represents the maximum change in peptide retention time relative to the random coil peptide C. Note that the higher the P_A_ value, the greater the self-association. It is very obvious that one “specificity determinant”, L7K, dramatically decreased peptide self-association from 12.61 min for d-dermaseptin S4 to 4.80 min for d-dermaseptin S4 L7K (Table 2). Adding a second “specificity determinant” at position 14 (A14K) further lowered the association parameter P_A_ value to 2.29 min for d-dermaseptin S4 L7K, A14K. The self-association is totally different for d-piscidin 1 and its analogs which all have very low P_A_ values (0.69~1.29) compared to d-dermaseptin S4 (Table 2).

### 3.6. Antibacterial Activity

Antibacterial activities against six strains of *P. aeruginosa* and eleven strains of *A. baumannii* are compared in Table 3. Geometric mean of MIC values was calculated to provide an overall view of antimicrobial activity of different analogs. It is clear that our peptides were effective in killing the microorganisms tested. Compared to a dramatic reduction in hemolytic activity, antibacterial activity of d-piscidin 1 and d-dermaseptin S4 analogs against eleven strains of *A. baumannii* maintained the same level (within 2-fold) upon the substitution of “specificity determinant(s)” (Table 3). 

### 3.7. Hemolytic Activity

**Figure 7:**
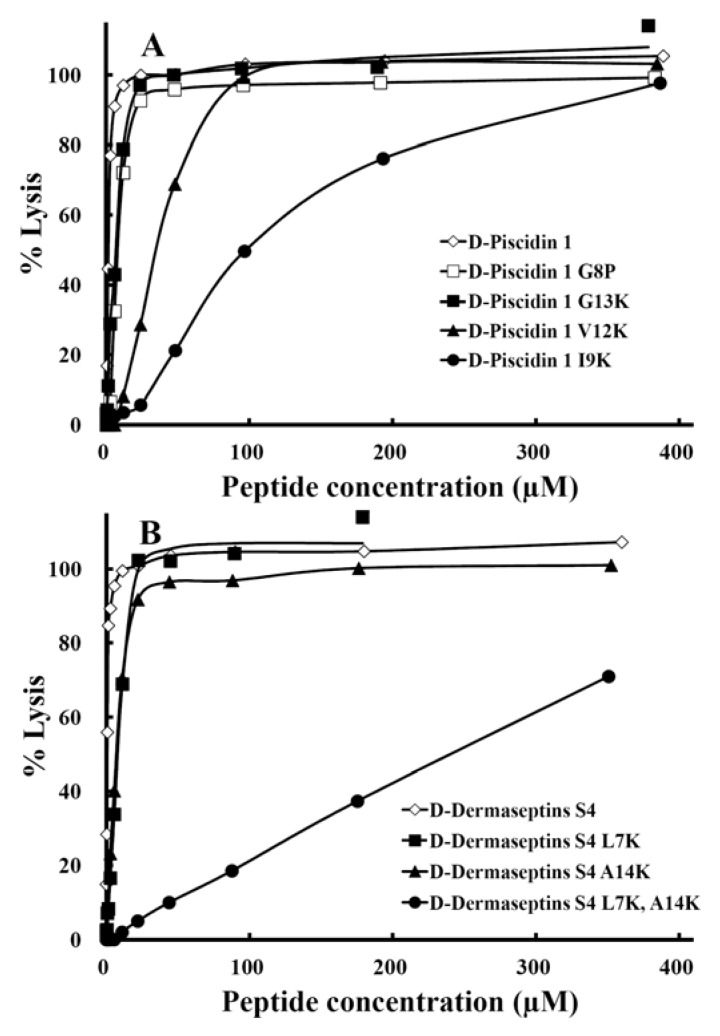
The hemolytic activity of peptide d-piscidin 1 and analogs (panel **A**) and d-dermaseptin S4 and analogs (panel **B**) after 18 hours of incubation time at 37 °C. The concentration-response curves of peptides for percentage lysis of human red blood cells (hRBC) are shown. The control for 100% hemolysis was a sample of erythrocytes treated with water. The peptide concentration is reported as µM.

The hemolytic activities of the peptides against human erythrocytes were determined as a measure of peptide toxicity toward higher eukaryotic cells. The effect of peptide concentration on erythrocyte hemolysis is shown in Figure 7. From these plots, the HC_50_ values, the peptide concentration that produces 50% hemolysis of human red blood cells after 18 hours in the standard microtiter dilution method was determined.

A single “specificity determinant” had a dramatic effect in lowering the hemolytic activity of d-piscidin 1 from HC_50_ value of 1.8 µM to 98 µM for d-piscidin 1 I9K, a 54-fold improvement (Table 4). Similarly, a single “specificity determinant” lowered the hemolytic activity of d-dermaseptin S4 from a HC_50_ value of 0.6 µM to 8.6 µM for d-dermaseptin S4 L7K, a 14-fold improvement (Table 4) and 7 µM for d-dermaseptin S4 A14K, an 11.7-fold improvement (Figure 7). The addition of a second “specificity determinant” in d-dermaseptin S4 to give the analog d-dermaseptin S4 L7K, A14K decreased the hemolytic activity from 0.6 µM to a HC_50_ value of 241µM, a 402-fold improvement in hemolytic activity. This also suggests a synergistic effect of having two “specificity determinants”. These two lysine residues also systematically lowered the self-association parameter (Figure 6).

Previous studies showed that the extreme toxicity of dermaseptin S4 is probably related to its higher hydrophobicity and self-association ability, as both nuclear magnetic resonance (NMR) and fluorescence methods have indicated that the peptide is in a high aggregation state in aqueous solutions [56], whereas sequential truncation of the N-terminal domain (hydrophobic stretch) of dermaseptin S4 confirmed that such hydrophobic interactions between the N-terminus of dermaseptin S4 monomers is primarily responsible for the peptide’s oligomerization in solution [23]. Self-association in solution is also probably responsible for limiting its spectrum of potential target cells [24]. The dramatic decrease in self-association of d-dermaseptin S4 L7K, A14K compared to native d-dermaseptin S4 correlates with the dramatic decrease in hemolytic activity of 402-fold (Table 4). 

Table 3Antimicrobial activity of d-piscidin 1 analogs and d-dermaseptin S4 analogs against *A. baumannii* (A) and *P. aeruginosa* (B) strains.

**Table:** **A. Antimicrobial activity against** ***Acinetobacter baumannii***

**Strain**		MIC ^a^ (µM)												Fold ^c^
		ATCC17978	ATCC19606	649	689	759	821	884	899	964	985	1012	GM ^b^	
**Peptide**	**Source**	Fatal meningitis	Urine	Blood	Groin	Gluteus	Urine	Axilla	Perineum	Throat	Pleural fluid	Sputum		
D-Piscidin 1		3.0	3.0	3.0	1.5	3.0	3.0	3.0	3.0	3.0	1.5	6.1	2.8	1.0
D-Piscidin 1 G13K		5.9	5.9	3.0	5.9	5.9	5.9	5.9	5.9	5.9	3.0	5.9	5.2	0.5
D-Piscidin 1 V12K		3.0	3.0	3.0	3.0	3.0	3.0	3.0	3.0	3.0	1.5	3.0	2.8	1.0
D-Piscidin 1 I9K		3.0	1.5	3.0	3.0	3.0	3.0	3.0	3.0	3.0	3.0	6.0	3.0	0.9
D-Dermaseptin S4		2.8	2.8	1.4	1.4	1.4	2.8	2.8	1.4	1.4	0.7	2.8	1.8	1.0
D-Dermaseptin S4 L7K		0.7	0.4	0.7	0.7	0.4	0.4	1.4	0.4	2.8	0.7	1.4	0.7	2.6
D-Dermaseptin S4 L7K,A14K		0.7	0.7	0.7	0.7	1.4	0.7	1.4	1.4	2.7	0.7	2.7	1.1	1.6

**Table:** **B. Antimicrobial activity against** ***Pseudomonas aeruginosa***

**Strain**		MIC ^a^ (µM)							Fold ^c^
		PAO1	PAK	PA14	CP204	M2	WR5	GM ^b^	
**Peptide**	**Source**	Human wound	—	—	Cystic fibrosis patient	Burn mouse model	Burn patient		
D-Piscidin 1		24.3	12.2	24.3	24.3	24.3	12.2	19.3	1.0
D-Piscidin 1 G13K		23.7	11.8	23.7	47.3	11.8	23.7	21.1	0.9
D-Piscidin 1 V12K		24.0	6.0	24.0	24.0	24.0	12.0	17.0	1.1
D-Piscidin 1 I9K		48.3	12.1	24.2	48.3	48.3	24.2	30.5	0.6
D-Dermaseptin S4		11.3	11.3	22.5	11.3	11.3	11.3	12.6	1.0
D-Dermaseptin S4 L7K		2.8	2.8	2.8	2.8	2.8	2.8	2.8	4.5
D-Dermaseptin S4 L7K,A14K		5.5	1.4	5.5	1.4	21.9	11.0	4.9	2.6

^a^ MIC is minimal inhibitory concentration (µM) that inhibited growth of different strains in Mueller-Hinton (MH) medium at 37 °C after 24 h. MIC is given based on three sets of determinations; ^b^ GM is the geometric mean of the MIC values from 11 different isolates of *A. baumannii* or 6 different isolates of *P. aeruginosa*; ^c^ The fold improvement in antimicrobial activity (geometric mean data) compared to that of native D-piscidin 1 or D-dermaseptin S4.

**Figure 8:**
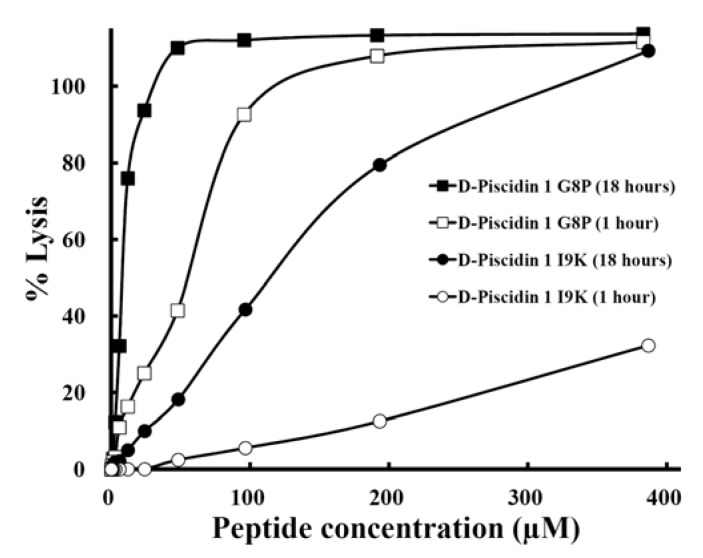
The comparison of hemolytic activity of peptide d-piscidin 1 G8P and d-piscidin 1 I9K after 1 hour or 18 hour treatment. The concentration-response curves of peptides for percentage lysis of human red blood cells (hRBC) are shown. The control for 100% hemolysis was a sample of erythrocytes treated with 0.1% Triton-X 100. The peptide concentration is in µM.

Hemolysis of human red blood cells is commonly used for *in vitro* assessment of AMP toxicity to normal cells. Many variations of this assay exist and inconsistency in red blood cells source, peptide exposure time and reporting of peptide concentrations impede comparison of AMP toxicity [57]. The length of time erythrocytes are exposed to AMPs during the hemolysis assay is the least standardized parameter of the method and the most commonly cited times are 30 min [58,59,60] and 1 hour [33,61,62,63]. However, higher exposure times are necessary to evaluate longer-term toxicity. For this reason, we compared hemolytic activity at 1 hour and 18 hours for two d-piscidin 1 analogs I9K, and G8P. As shown in Figure 8, the HC_50_ value increased dramatically from 1 hour to 18 hours. Thus, the hemolytic activity (HC_50_) for the G8P analog at 1 hour incubation time at 37 °C was 55 µM; in contrast, at 18 hours at 37 °C, the HC_50_ was 8 µM (a 7-fold increase in hemolytic activity from 1 to 18 hours). Similarly, for d-piscidin 1 I9K, the hemolytic activity at 1,000 µg/mL (387 µM) for 1 hour was only 32% hemolysis (HC_50_ could not be determined) and at 18 hour incubation time the HC_50_ was 115 µM. Based on these results it is clear that when determining hemolytic acitivity of antimicrobial peptides concentration should be varied up to at least 1,000 µg/mL in the assay and an exposure/incubation time with human red blood cells should be 18 hours. Under these conditions, HC_50_ values can be easily and accurately determined and will provide the best data for selecting analogs with low or no hemolytic activity. Using 30 min or 1 h exposure times and lower peptide concentrations can provide misleading data.

**Table 4:** Summary of biological activity of d-piscidin 1 and d-dermaseptin S4 analogs.

Peptide Name	Hemolytic activity		Antimicrobial activity					
			*Acinetobacter baumannii*			*Pseudomonas aeruginosa*		
	HC_50_^a^ (µM)	Fold ^b^	MIC_GM_^c^ (µM)	T. I. ^d^	Fold ^e^	MIC_GM_^c^ (µM)	T. I. ^d^	Fold ^e^
d-Piscidin 1	1.8	1.0	2.8	0.6	1.0	19.3	0.1	1.0
d-Piscidin 1 G13K	7.0	3.9	5.2	1.3	2.2	21.1	0.3	3.0
d-Piscidin 1 V12K	35	19	2.8	13	22	17.0	2.1	21
d-Piscidin 1 I9K	**98**	**54**	3.0	**33**	**55**	30.5	**3.2**	**32**
d-Dermaseptin S4	0.6	1.0	1.8	0.3	1.0	12.6	0.05	1.0
d-Dermaseptin S4 L7K	8.6	14	0.7	12	40	2.8	3.1	62
d-Dermaseptin S4 L7K,A14K	**241**	**402**	1.1	**219**	**730**	4.9	**49**	**980**

^a^ HC_50_ is the concentration of peptide (µM) that results in 50% hemolysis after 18 h at 37 °C. The analogs with the best HC_50_ values are bolded; ^b^ The fold improvement in HC_50_ compared to that of d-piscidin 1 or d-dermaseptin S4. The analogs with the best fold improvements are bolded; ^c^ MIC is the minimum inhibitory concentration (µM) of peptide that inhibits growth of bacteria after 24 h at 37 °C. MIC_GM_ is the geometric mean of the MIC values from 11 different isolates of *A. baumannii* or 6 different isolates of *P. aeruginosa*; ^d^ T.I. denotes therapeutic index, which is the ratio of the HC_50_ value (µM) over the geometric mean MIC value (µM). Large values indicate greater antimicrobial specificity. The analogs with the best therapeutic indices are bolded; ^e^ The fold improvement in therapeutic index compared to that of d-piscidin 1 or d-dermaseptin S4. The analogs with the best fold improvements are bolded.

### 3.8. Therapeutic Index

The therapeutic indices are shown in Table 4. Large values indicate greater antimicrobial specificity. The substitution of Lys residues in the center of the non-polar face maintained antimicrobial activity against six strains of *P. aeruginosa* and eleven strains of *A. baumannii* and dramatically decreased hemolytic activities against human red blood cells by 54-fold and 402-fold for d-piscidin 1 I9K and d-dermaseptin S4 L7K, A14K, respectively. The geometric mean of the MIC values from eleven different diverse strains of *A. baumannii* was unchanged between native d-piscidin 1 and d-piscidin I9K at 2.8 µM and 3.0 µM, respectively. The geometric mean of the MIC values from six different diverse strains of *P. aeruginosa* between native d-piscidin 1 and d-piscidin I9K increased from 19.3 µM and 30.5 µM. In the case of d-dermaseptin S4, the geometric mean of MIC values for *A. baumannii* decreased from 1.8 µM for d-dermaseptin S4 to 1.1 µM for d-dermaseptin S4 L7K, A14K indicating a small improvement in antimicrobial activity. Similarly, with *P. aeruginosa*, the improvement in antimicrobial activity changed from a geometric mean of 12.6 µM for d-dermaseptin S4 to 4.9 µM for d-dermaseptin S4 L7K, A14K, representing a 2.5-fold improvement. d-piscidin 1 I9K, the most selective peptide among piscidin 1 analogs, showed an increase in the therapeutic index from 0.1 for native d-piscidin 1 to 3.2 for *P. aeruginosa*, a 32-fold improvement; while for *A. baumannii*, the therapeutic index increased from 0.6 for native l-piscidin 1 to 33, a 55-fold improvement. d-dermaseptin S4 L7K, A14K, the most selective peptide of dermaseptin S4 analogs, showed a dramatically improved therapeutic index of 980-fold for *P. aeruginosa*, from 0.05 for native d-dermaseptin S4 to 49 for this analog; for *A. baumannii*, the therapeutic index improved by 730-fold from 0.3 for native d-dermaseptin S4 to 219 for this analog.

### 3.9. Mechanism of AMP Interaction with Membranes

The above observations can be explained by our membrane discrimination mechanism [13,16,37]. We suggest that AMPs have activity against zwitterionic eukaryotic membranes by a pore-formation mechanism (“barrel-stave” mechanism [64,65]): the peptides must be able to form a transmembrane pore. However the introduction of “specificity determinant(s)” prevents transmembrane penetration in the bilayer of eukaryotic cells. On the other hand, interaction of AMPs with negatively charged prokaryotic cell membranes utilizes the detergent-like mechanism (“carpet” mechanism [66]) and transmembrane insertion is not required for antimicrobial activity. The peptides can lie parallel to the membrane surface where the positively charged residues on the polar face interact with the negatively charged phospholipid head groups of the bilayer and the ε-amino group of the Lys side-chain of the “specificity determinant(s)” may be long enough to avoid the hydrophobicity of the bilayer when lying parallel to the membrane surface even though they are on the non-polar face of the AMP. The peptides are still able to disrupt the lipid bilayer causing cytoplasmic leakage and cell death.

The differences in how piscidin 1 interacts with the two membrane types (zwitterionic *vs.* negatively-charged) were suggested by the previous NMR structures, CD data and MD simulations [67,68]. Results strongly demonstrate that the peptide inserts perpendicular to the POPC (1-palmitoyl-oleoyl-glycero-phosphocholine, neutral lipid) bilayer , whereas the peptide interacts only peripherally with the POPG (palmitoyl-oleoylphosphtidylglycerol, negatively-charged lipid) bilayer (a carpet-like manner) [67]. Furthermore, the hydrophobic residues largely interact with the zwitterionic membrane model while positively charged residues favorably interact with the negatively-charged membranes [67]. The results of De Angelis *et al.* [68] suggest that not only does the cationic peptide preferentially interact with the negatively charged lipid molecules, but it may also cluster them; such peptide-lipid interactions are optimized at the bilayer interface, possibly as a prerequisite for bilayer disruption which allows piscidin to initiate its disruptive behavior in the form of small aggregates. In conclusion, (1) zwitterionic and negatively-charged phospholipids did not have the same response against piscidin 1 binding; (2) such differential interactions are related to the balance of electrostatic and hydrophobic interactions of piscidin 1 with the zwitterionic *vs.* negatively-charged bilayer types [67].

## 4. Conclusions

We have taken two examples of native AMPs, piscidin 1 and dermaseptin S4, to demonstrate the universality of our “specificity determinant” design concept to effect a dramatic reduction in AMP toxicity (measured by hemolytic activity of human red blood cells). Substitution of a single lysine residue in the center of the non-polar face of d-piscidin 1 lowered the hemolytic activity by 54-fold from a HC_50_ value of 1.8 µM to 98 µM for d-piscidin 1 I9K. In the case of d-dermaseptin S4, substitution of two lysine residues in the center of the non-polar face lowered the hemolytic activity by 402-fold from a HC_50_ value of 0.6 µM to 241 µM for d-dermaseptin S4 L7K, A14K. Antimicrobial activity, as expressed by the geometric mean of 11 diverse strains of *A. baumannii*, was maintained for d-piscidin 1 I9K and a small improvement was observed for d-dermaseptin S4 L7K, A14K. Improvements in the therapeutic indices for these analogs were 55-fold (d-piscidin 1 I9K) and 730-fold (d-dermaseptin S4 L7K, A14K). Similarly, improvements in the therapeutic indices against 6 diverse strains of *P. aeruginosa* for these analogs were 32-fold and 980-fold, respectively. Comparison of the therapeutic indices of these two analogs (summarized in Table 4) showed that d-dermaseptin S4 L7K, A14K, with a therapeutic index of 49 for *P. aeruginosa* and 219 for *A. baumannii*, has the desired properties for potential systemic use as a therapeutic against these two Gram-negative pathogens. In addition to the desired biological activity, d-dermaseptin S4 L7K, A14K has the desired biophysical properties. The two specificity determinants dramatically decreased helicity in aqueous medium, decreased overall hydrophobicity and hydrophobicity of the non-polar face, decreased amphipathicity and decreased self-association, all of which keeps the peptide as a random coil (no structure) in aqueous medium. This result supports our mechanism of easy passage of the unstructured peptide monomer through the capsule and cell wall to reach the cytoplasmic membrane, the target of the AMP. At the membrane surface, the peptide cannot form a transmembrane pore due to the presence of the positively-charged lysine residues on the non-polar face which prevents transmembrane burial in the bilayer. Thus, the AMP cannot enter the membrane of eukaryotic cells but can lie perpendicular to the membrane surface in the interface region of prokaryotic cells, where the hydrophobicity of the lipid bilayer induces the AMP into its α-helical structure and the AMP can disrupt the lipid bilayer by the carpet mechanism, causing leakage and cell death.

Finally, we have clearly shown that the use of short exposure time (1 h) for determining hemolytic activity may underestimate the hemolytic activity of AMPs and that an 18 h exposure and peptide concentrations up to 1000 µg/mL may be used for selecting analogs with improved therapeutic indices.
